# Failure patterns after adjuvant chemoradiotherapy following endoscopic resection for superficial esophageal squamous cell carcinoma

**DOI:** 10.1002/cam4.2365

**Published:** 2019-06-20

**Authors:** Toshiki Ikawa, Ryu Ishihara, Koji Konishi, Masahiro Morimoto, Takero Hirata, Naoyuki Kanayama, Sachiko Yamamoto, Noriko Matsuura, Kentaro Wada, Kenji Hayashi, Kazuhiko Ogawa, Teruki Teshima

**Affiliations:** ^1^ Department of Radiation Oncology Osaka International Cancer Institute Osaka Japan; ^2^ Department of Gastrointestinal Oncology Osaka International Cancer Institute Osaka Japan; ^3^ Department of Radiation Oncology Osaka University Graduate School of Medicine Osaka Japan

**Keywords:** chemoradiotherapy, endoscopic resection, esophageal neoplasms, squamous cell carcinoma

## Abstract

**Background:**

This study evaluated the locations of lymph node recurrence and their association with irradiation fields used for radiotherapy after adjuvant chemoradiotherapy following endoscopic resection for superficial esophageal squamous cell carcinoma.

**Methods:**

Medical records of 96 consecutive patients with superficial esophageal squamous cell carcinoma who underwent adjuvant chemoradiotherapy following endoscopic resection were reviewed. Computed tomography was used to identify whether nodal recurrences were within the elective nodal irradiation field. The cumulative incidence of recurrence was calculated, accounting for death as a competing risk. Univariate and multivariate analyses identified factors predicting nodal recurrence.

**Results:**

The median follow‐up period was 61 months (range: 6‐137 months). Seven patients (7.3%) developed lymph node recurrence only; two patients (2.1%) developed nodal plus local recurrence. Six of the seven cases without local recurrence involved the elective nodal irradiation field, with five cases involving the recurrent nerve lymph nodes. The 5‐year cumulative incidence of lymph node recurrence was higher for T1b tumors with lymphovascular invasion than for T1a tumors with lymphovascular invasion (17.6% vs 6.2%, *P *=* *0.086; HR: 3.74, 95% CI: 0.80‐17.52, *P *=* *0.094) and T1b tumors without lymphovascular invasion (17.6% vs 3.3%, *P *=* *0.031; HR: 6.78, 95% CI: 0.80‐57.63, *P *=* *0.080).

**Conclusions:**

Lymph node recurrence frequently involved the elective nodal irradiation field, with recurrent nerve lymph nodes being common metastasis sites. The high incidence of nodal recurrence for T1b tumors with lymphovascular invasion highlights a need for new strategies for treating this subset of superficial esophageal squamous cell carcinomas.

## 1 INTRODUCTION

Esophageal squamous cell carcinoma accounts for approximately 90% of esophageal cancers in eastern Asia, including China and Japan.^1^, ^2^ Better diagnostic modalities, such as multimodal endoscopic imaging, have increased the reported incidence of superficial esophageal cancer (SEC).^3^, ^4^, ^5^ Cases of SEC are identified based on the depth of invasion (DOI) limited to the mucosa or submucosa, regardless of lymph node or distant organ metastasis.^6^, ^7^

There are three main treatments for SEC: esophagectomy, definitive chemoradiotherapy (CRT), and endoscopic resection (ER). Esophagectomy has been a standard treatment, although potential postoperative complications preclude surgery for older patients or those with certain comorbidities.^8^ Definitive CRT has been a standard treatment for superficial esophageal squamous cell carcinoma (SESCC) in patients who decline or cannot tolerate surgery. However, residual tumor or local recurrence after definitive CRT can be problematic, as salvage esophagectomy may be associated with high rates of morbidity and mortality.^9^ Thus, endoscopic mucosal resection (EMR) and endoscopic submucosal resection (ESD) are widely used as curative and less invasive options for SEC, especially in Japan.^2^ The indication for ER is usually mucosal (T1a) SEC, which has a low risk of lymph node metastasis. However, there is controversy regarding the use of ER for submucosal (T1b) SEC, as the submucosal invasion is associated with an increased risk of lymph node metastasis.^10^, ^11^, ^12^

The combination of ER and adjuvant CRT has emerged as a new strategy for treating SESCC,^13^, ^14^, ^15^, ^16^ as ER plus adjuvant CRT can provide a lower incidence of residual tumors or local recurrence than definitive CRT. Furthermore, pre‐CRT ER may help predict the risk of metastasis and guide subsequent treatment.^14^, ^17^ In our institution, ER has been widely performed for both T1b and T1a SESCC, and adjuvant CRT with elective nodal irradiation (ENI) has been performed to reduce the risk of locoregional recurrence when the pathological examination reveals high‐risk metastatic features, such as T1b DOI or the presence of lymphovascular invasion (LVI).^12^, ^14^, ^17^ Hamada et al^14^ reported the efficacy and safety of this combination therapy for SESCC at our institution, based on good survival rates after 3 years (87%) and 5 years (75%), with low rates of local recurrence from resection sites (3%). Nevertheless, metastatic recurrences were observed in 16.7% of tumors with LVI, typically involving lymph node recurrence (LNR). As radiotherapy is intended to eliminate micrometastases within the irradiation field, further evidence is needed to confirm whether radiotherapy in this strategy can reduce the risk of LNR. Although several retrospective studies have shown the efficacy and safety of ER plus adjuvant CRT for SESCC,^13^, ^14^, ^15^, ^16^ few have evaluated the LNR locations based on the radiation field. Therefore, this study evaluated failure patterns among patients who underwent ER plus adjuvant CRT for SESCC, in order to determine whether fields of radiotherapy or other factors predicted the incidence of LNR.

## 2 METHODS

### 2.1 Patients

This retrospective review was approved by our institutional review board. Consecutive patients with esophageal cancer who received adjuvant CRT following ER at our institution between January 2006 and December 2014 were evaluated. Inclusion criteria were: clinical T1N0M0 esophageal cancer (UICC TNM classification, 7th edition), histologically proven squamous cell carcinoma, histologically proven T1b DOI or presence of LVI, receipt of adjuvant CRT with ENI following ER, and follow‐up of ≥3 months. The exclusion criteria were failure to complete the radiotherapy and the administration of only radiotherapy. Patients were classified into T1a DOI plus LVI (T1aLVI+), T1b DOI but no LVI (T1bLVI−), and T1b DOI plus LVI (T1bLVI+) subgroups. Clinical staging was based on endoscopy and computed tomography (CT) of the neck, chest, and abdomen. Endoscopic ultrasonography and ^18^F‐FDG PET‐CT were performed as necessary. Clinically malignant lymph nodes in the recurrent nerve region were identified using the CT findings based on the shortest diameter of 0.5 cm or other cervical, mediastinal, and abdominal lymph nodes with the shortest diameter of 1.0 cm.

### 2.2 Endoscopic resection and pathological examination

ER was performed either through EMR or ESD. Pathological examination of resected specimens was performed according to the Japanese Classification of Esophageal Cancer,^6^, ^7^ which includes the tumor size, histological subtypes, DOI, LVI, and resection margin status. Based on the UICC TNM classification (7th edition), DOI was classified as T1a (tumor invading the lamina propria or muscularis mucosae) or T1b (tumor invading the submucosa). Furthermore, based on the Japanese Classification of Esophageal Cancer, T1b cases were classified as T1b‐SM1 (tumor invading the submucosa to a depth of ≤200 μm) or T1b‐SM2 (tumor invading the submucosa to a depth of >200 μm).^6^, ^7^

### 2.3 Chemoradiotherapy

**Figure 1:**
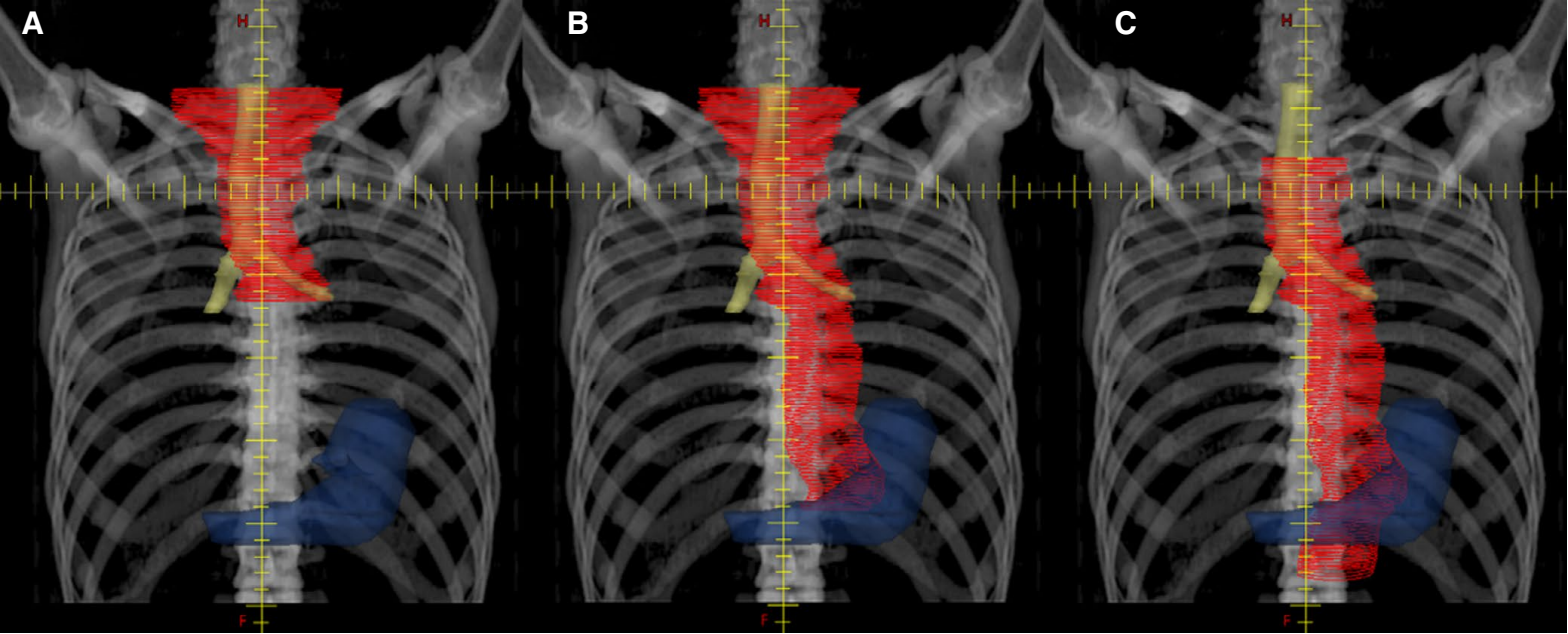
The typical clinical target volume (CTV) for elective nodal irradiation (ENI) based on tumor location. The contours in red, yellow, and blue represent the contours of the CTV, trachea plus primary bronchi, and stomach, respectively. A, The CTV for upper thoracic esophageal cancer generally encompassed the region from the bilateral supraclavicular, cervical paraesophageal, and mediastinal lymph nodes to the tracheal bifurcation. B, The CTV for middle thoracic esophageal cancer generally encompassed the bilateral supraclavicular, cervical paraesophageal, mediastinal, paracardial, lesser curvature, and left gastric lymph nodes. C, The CTV for lower thoracic esophageal cancer generally encompassed the mediastinal, paracardial, lesser curvature, left gastric, and celiac artery lymph nodes

Adjuvant CRT was started after confirming ER‐induced ulcer healing. Patients were treated using three‐dimensional conformal radiotherapy, and the clinical target volume (CTV) for ENI was defined as that including the regional lymph nodes based on the tumor's location (Figure 1). The prescribed dose was 1.8‐2.0 Gy/day, administered 5 days per week to a total dose of 40‐41.4 Gy. When pathological examination of the resected specimen revealed a positive vertical margin, or when malignancy was suspected based on mildly swollen lymph nodes on the radiotherapy planning CT images, a boost dose of 9.0‐20.0 Gy in 5‐10 fractions was delivered to the resection site or to the lymph nodes suspected of metastasis. The concurrent chemotherapy usually consisted of cisplatin (70 mg/m^2^/day on days 1 and 29) plus 5‐fluorouracil (700 mg/m^2^/day as a continuous infusion on days 1‐4 and days 29‐32), although the doses were reduced if necessary based on the patient's condition.

### 2.4 Follow‐up and failure patterns

Follow‐up examinations generally consisted of gastrointestinal endoscopy and CT scans of the neck, chest, and abdomen every 4 months up to 2 years posttreatment and every 6 months thereafter. Biopsy and/or PET‐CT were performed if necessary. Failure patterns were defined based on the first site of recurrence, although mucosal recurrence that could be removed by ER was not counted. Local recurrence was defined as recurrence within the esophagus, including recurrent primary and new metachronous esophageal cancers. The definition of LNR was any recurrence in any lymph node area, including the regional and distant lymph nodes. Distant recurrence was defined as metastasis to distant organs. The locations of LNR were classified according to the Japanese Classification of Esophageal Cancer,^6^, ^7^ and involvement of the ENI field was examined by comparing the current CT or PET‐CT results to the treatment plan. The cumulative incidence of LNR was counted regardless of prior local or distant recurrences.

### 2.5 Statistical analysis

Overall survival (OS) was defined as the time from the start of CRT to death from any cause. Progression‐free survival (PFS) was defined as the time from the start of CRT until recurrence or death from any cause. The OS and PFS rates were calculated using the Kaplan‐Meier method. The cumulative incidence of LNR was calculated using the cumulative incidence function and accounting for death without LNR as a competing risk. Differences in this outcome according to clinical factors were assessed using Gray's test.^18^ Univariate and multivariate analyses were performed using Fine and Gray's proportional subhazards model to identify factors that predicted LNR.^19^ Two‐sided *P*‐values of <0.05 were considered statistically significant; values of ≥0.05 to <0.1 were considered marginally significant. All statistical analyses were performed using EZR (Saitama Medical Center, Jichi Medical University, Saitama, Japan).^20^

## 3 RESULTS

### 3.1 Patient, tumor, and treatment characteristics

This study included 96 patients; Table 1 shows the patient, tumor, and treatment characteristics. The 5‐year OS and PFS rates for all patients were 82.4% (95% confidence interval [CI]: 73.0%‐88.9%) and 78.5% (95% CI: 69.0%‐85.8%), respectively. The patients were predominantly male (n = 89, 92.7%), with a median age of 67 years (range: 42‐82 years). The middle thoracic esophagus was the most common primary tumor location (n = 53, 55.2%), followed by the lower thoracic esophagus (n = 27, 28.1%). The patients underwent ESD (n = 77, 80.2%) or EMR (n = 19, 19.8%), and the pathological examinations revealed DOIs corresponding to T1a (n = 32, 33.3%), T1b‐SM1 (n = 12, 12.5%), and T1b‐SM2 (n = 52, 54.2%), with LVI in 66 cases (68.8%) and a positive vertical margin in eight (8.3%).

**Table 1:** Patient, tumor, and treatment characteristics (n = 96)

Age, y		
Median	67	
Range	42‐82	
Sex, n (%)		
Male	89	(92.7)
Female	7	(7.3)
Tumor location, n (%)		
Cervix	2	(2.1)
Upper thorax	14	(14.6)
Middle thorax	53	(55.2)
Lower thorax	27	(28.1)
Endoscopic resection, n (%)		
ESD	77	(80.2)
EMR	19	(19.8)
Tumor size, mm		
Median	25	
Range	5‐75	
Depth of invasion, n (%)		
T1a	32	(33.3)
T1b‐SM1	12	(12.5)
T1b‐SM2	52	(54.2)
Lymphovascular invasion, n (%)^a^		
Negative	30	(31.3)
Positive	66	(68.8)
Vertical resection margin, n (%)		
Negative	88	(91.7)
Positive	8	(8.3)
Chemotherapy, n (%)		
Cisplatin+ 5‐fluorouracil	93	(96.9)
Others^b^	3	(3.1)
Radiation dose, n (%)		
40 or 41.4 Gy	86	(89.6)
50 or 50.4 Gy	9	(9.4)
60 Gy	1	(1.0)

Abbreviations: ESD, endoscopic submucosal resection; EMR, endoscopic mucosal resection; T1b‐SM1, tumor invading the submucosa to a depth of ≤200 μm; T1b‐SM2, tumor invading the submucosa with a depth of >200 μm.

a Percentages in this column do not add up to exactly 100% because of rounding.

b Others include docetaxel, cisplatin, and cisplatin + S‐1.

Table 2 shows the relationship between DOI and LVI, with the lesions classified as T1aLVI+ (n = 32, 33.3%), T1bLVI− (n = 30, 31.3%), or T1bLVI+ (n = 34, 35.4%). Almost all patients (n = 93, 96.9%) received chemotherapy consisting of cisplatin plus 5‐fluorouracil; only three patients (3.1%) received docetaxel, cisplatin, or cisplatin plus S‐1. Based on a positive vertical margin and suspected metastasis (mildly enlarged lymph nodes on the radiotherapy planning CT images), some patients received additional doses of 9‐20 Gy to the resected site (n = 8, 8.3%) or lymph nodes suspected of metastasis (n = 2, 2.1%).

**Table 2:** Relationship between depth of invasion and lymphovascular invasion^a^

DOI	T1a	T1b		T1b‐All
		T1b‐SM1	T1b‐SM2	
LVI				
Negative		10 (10.4%)	20 (20.8%)	30 (31.3%)
Positive	32 (33.3%)	2 (2.1%)	32 (33.3%)	34 (35.4%)

Abbreviations: DOI, depth of invasion; LVI, lymphovascular invasion; T1b‐SM1, tumor invading the submucosa to a depth of ≤200 μm; T1b‐SM2, tumor invading the submucosa with a depth of >200 μm.

a Percentages in this table do not add up to exactly 100% because of rounding.

### 3.2 Failure patterns

Twenty‐six patients (27.1%) developed recurrence during a median follow‐up of 61 months (range: 6‐137 months); the failure patterns are summarized in Table 3. However, 11 cases (11.5%) involved mucosal recurrences that were successfully removed by ER and were not counted as recurrence. Among the remaining 15 cases (15.6%), local recurrence was detected in eight (8.3%), three of which were accompanied by lymph node or distant organ metastasis, with a median time to recurrence of 31 months (range: 6‐81 months). Among the eight local recurrences, four cases involved tissue near or at the resection site (ie, recurrence of primary esophageal cancer). Among the eight patients who developed local recurrence, six patients received salvage treatment, including surgery and ESD, although four patients subsequently experienced multiple lymph node or distant metastases.

**Table 3:** Patterns of first recurrence in 96 patients

Location of first recurrence^a^	Patients, n	Median time to recurrence, mo (range)
Local^b^		
Local only	5	28 (6‐67)
Local + lymph node	2	11, 34
Local + distant	1	81
Lymph node only^c^	7	25 (16‐70)
Distant only^d^	0	Not available
Total	15	28 (6‐81)

a Mucosal recurrence removed by endoscopic resection is not counted as recurrence.

b Local recurrence is defined as recurrence within the esophagus, including recurrent primary and new metachronous esophageal cancer.

c Lymph node recurrence is defined as recurrence in any lymph node areas, including regional and distant lymph nodes.

d Distant recurrence is defined as metastasis to distant organs.

Seven patients (7.3%), all of whom received ESD before CRT, experienced solely LNR, with a median time to recurrence of 25 months (range: 16‐70 months). The locations of cases solely involving LNR and the tumor characteristics are summarized in Table 4. All seven lesions had a T1b DOI, six lesions were LVI+, five cases only involved a solitary LNR, and six cases involved the ENI. The most common LNR location was the right or left recurrent nerve lymph nodes (n = 5), followed by the supraclavicular lymph nodes (n = 2). Among the seven patients who solely developed LNR, six patients received salvage surgery or radiotherapy/CRT, although they all subsequently experienced multiple lymph node and distant metastases.

**Table 4:** Tumor characteristics when the first recurrence was solely lymph node recurrence

Tumor location	ER	Size, mm	DOI	LVI	Vertical resection margin	Dose, Gy	Time to recurrence, mo	Location of lymph node recurrence^a^	Solitary recurrence	Inside field of ENI
Ut	ESD	13	T1b‐SM2	+	−	40	19	#106recL	Yes	Yes
Ut	ESD	30	T1b‐SM2	+	−	40	70	#106recR	Yes	Yes
Mt	ESD	15	T1b‐SM2	−	−	40	37	#106recR	Yes	Yes
Mt	ESD	15	T1b‐SM1	+	−	40	24	#106recR, #104	No	Yes to all
Mt	ESD	44	T1b‐SM2	+	−	41.4	25	#106recL	Yes	Yes
Mt	ESD	12	T1b‐SM2	+	+	50	16	#1	Yes	Yes
Mt	ESD	9	T1b‐SM2	+	−	40	40	#104, #100ac, #ALNs	No	No to all

Abbreviations: #1, right paracardial lymph nodes; #100ac, accessory nerve lymph nodes; #104, supraclavicular lymph nodes; #106recL, left recurrent nerve lymph nodes; #106recR, right recurrent nerve lymph nodes; #ALNs, axillary lymph nodes; DOI, depth of invasion; ENI, elective nodal irradiation; ER, endoscopic resection; ESD, endoscopic submucosal resection; Lt, lower thoracic; LVI, lymphovascular invasion; Mt, middle thoracic; T1b‐SM1, tumor invading the submucosa to a depth of ≤200 μm; T1b‐SM2, tumor invading the submucosa with a depth of >200 μm; Ut, upper thoracic.

a The location of lymph node recurrence is classified according to the Japanese Classification of Esophageal Cancer.^6^, ^7^

### 3.3 Cumulative incidence and predictors of LNR

**Figure 2:**
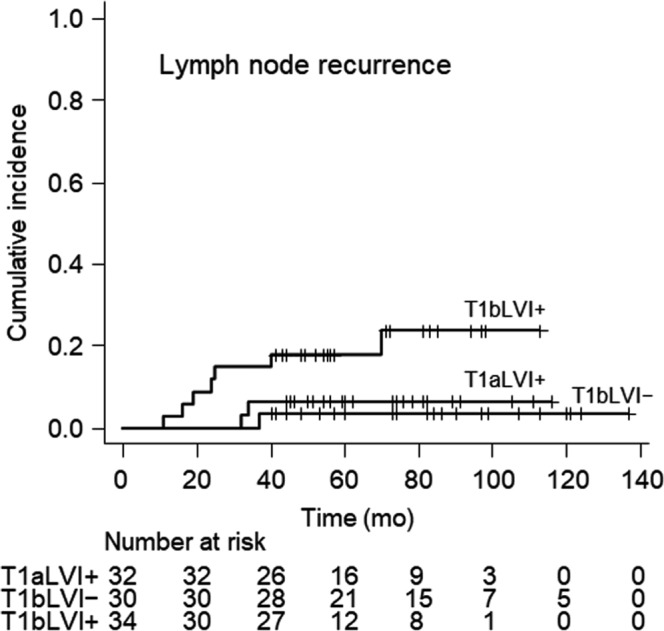
The cumulative incidence of lymph node recurrence stratified according to the depth of invasion and lymphovascular invasion (LVI). The cumulative incidence of lymph node recurrence was marginally higher for T1b tumors with LVI than for T1a tumors with LVI (T1bLVI+ vs T1aLVI+, *P *=* *0.086) and was significantly higher for T1bLVI + tumors than for T1b tumors without LVI (T1bLVI + vs T1bLVI–, *P *=* *0.031)

The 3‐year and 5‐year cumulative incidences of LNR were 7.3% (95% CI: 3.2%‐13.7%) and 9.4% (95% CI: 4.6%‐16.3%), respectively. Figure 2 shows the cumulative incidence curves for LNR according to the DOI‐ and LVI‐based groups (T1aLVI+, T1bLVI−, and T1bLVI+). The cumulative incidence of LNR was marginally higher for T1bLVI+ tumors than for T1aLVI+ tumors (*P *=* *0.086) and significantly higher than that for T1bLVI− tumors (*P *=* *0.031). The 5‐year cumulative incidences of LNR were 6.2% for T1aLVI+ tumors (95% CI: 1.1%‐18.4%), 3.3% for T1bLVI− tumors (95% CI: 0.2%‐14.8%), and 17.6% for T1bLVI+ tumors (95% CI: 7.0%‐32.2%). Univariate and multivariate analyses were performed using age, tumor location, tumor size, vertical resection margin status, and the DOI‐ and LVI‐based risk groups as variables. Multivariate analysis revealed that the DOI‐ and LVI‐based groups might predict the risk of LNR (Table 5), which was marginally higher for T1bLVI+ tumors than for T1aLVI+ (hazard ratio [HR]: 3.74, 95% CI: 0.80‐17.52, *P *=* *0.094) and T1bLVI− tumors (HR: 6.78, 95% CI: 0.80‐57.63, *P *=* *0.080).

**Table 5:** Independent predictors of lymph node recurrence

	Univariate analysis			Multivariate analysis		
	HR	95% CI	*P‐*value	HR	95% CI	*P‐*value
Age						
>65 vs ≤65 y	1.59	0.42‐6.01	0.50	1.74	0.44‐6.89	0.43
Tumor location						
Ce & Ut vs Lt	5.19	0.54‐50.21	0.16	3.35	0.35‐32.17	0.29
Mt vs Lt	3.11	0.36‐26.64	0.30	3.14	0.31‐32.19	0.34
Tumor size						
>30 vs ≤30 mm	0.80	0.21‐3.05	0.74	0.92	0.18‐4.64	0.92
DOI and LVI						
T1bLVI+ vs T1aLVI+	3.61	0.77‐16.92	0.10	3.74	0.80‐17.52	0.094
T1bLVI+ vs T1bLVI–	7.20	0.90‐57.84	0.063	6.78	0.80‐57.63	0.080
Vertical resection margin						
Positive vs negative	1.34	0.16‐11.16	0.79	1.10	0.11‐11.01	0.94

Abbreviations: Ce, cervical esophagus; CI, confidence interval; DOI, depth of invasion; HR, hazard ratio; Lt, lower thoracic; LVI, lymphovascular invasion; Mt, middle thoracic; T1aLVI+, T1a tumor with lymphovascular invasion; T1bLVI+, T1b tumor with lymphovascular invasion; T1bLVI−, T1b tumor without lymphovascular invasion; Ut, upper thoracic.

## 4 DISCUSSION

We analyzed the locations of LNR after adjuvant CRT following ER to investigate the association between the irradiation field and LNR. Interestingly, our results indicate that most lesions involving solely LNR occurred inside of the ENI field, with the right or left recurrent nerve lymph nodes being the most frequent site of metastasis in those cases. In addition, most solely LNR cases involved a solitary lesion. In this context, recurrent nerve lymph nodes are well known as frequent metastasis sites of thoracic esophageal squamous cell carcinoma.^21^, ^22^, ^23^, ^24^ This is related to the submucosal lymphatic vessels running longitudinally along the thoracic esophagus, directly draining into the proximal and distal ends and consequently connecting the recurrent nerve and perigastric lymph nodes.^25^ Therefore, the locations of lymph node metastasis of SESCC exhibit a unique distribution to the recurrent nerve or perigastric lymph nodes, which can involve a solitary lymph node.^12^, ^21^, ^24^, ^26^ The perigastric lymph nodes are also known as frequent metastasis sites, especially for middle and lower thoracic esophageal cancer.^21^, ^24^, ^26^ For example, Matsubara et al^21^ retrospectively investigated pathological findings from esophagectomy with lymph node dissection, which revealed solitary lymph node involvement in 52% of T1 tumors with lymph node metastasis. Furthermore, among T1‐2 tumors with solitary lymph node metastasis, the recurrent nerve lymph nodes were the most frequent sites of metastasis, followed by the perigastric lymph nodes. Thus, the CTV of the ENI in the present study was determined to involve the recurrent nerve lymph nodes for all locations of esophageal cancer and the perigastric lymph nodes for middle and lower thoracic esophageal cancer. Given the low incidence of LNR outside the ENI, the determination of the CTV based on tumor location appears to be appropriate.

We estimated the cumulative incidence of LNR and performed univariate and multivariate analyses to identify factors that predicted LNR. The 5‐year cumulative incidences of LNR were 6.2% for T1aLVI+ tumors, 3.3% for T1bLVI− tumors, and 17.6% for T1bLVI+ tumors. Multivariate analysis revealed that T1bLVI+ status was marginally associated with a higher risk of LNR than T1aLVI+ or T1bLVI− status. Other surgeons have also reported that the incidence of pathological lymph node metastasis was 4.3%‐15.4% for T1a tumors and 22.5%‐39.3% for T1b tumors.^10^, ^11^, ^12^, ^27^ Thus, the incidence from the present study tended to be lower than the previously reported incidences for these tumors. This finding suggests that adjuvant CRT may help reduce the incidence of LNR in T1aLVI+ and T1b tumors. However, the incidence in T1bLVI+ tumors remains high, and the prognosis after LNR was poor, although salvage surgery or radiotherapy/CRT was performed in most patients who experienced solely LNR. Our result of the poor prognosis after LNR is in agreement with that of Jingu et al who showed the outcome of reirradiation for lymph node oligo‐recurrence from esophageal cancer.^28^ Therefore; further strategies are needed to develop more effective treatments to prevent LNR.

As LNR frequently occurred at specific sites within the ENI field, we hypothesize that macrometastasis may already exist in sites with a high risk of LNR, such as the recurrent nerve or perigastric lymph nodes, which would indicate that the prescribed dose to the ENI was insufficient to control the macrometastasis. Although various modalities, including CT and PET‐CT, are used to detect macrometastasis before starting treatment for esophageal cancer, they do not appear to be sensitive enough to identify small metastasis.^29^, ^30^ However, in contrast with our results, Uchinami et al reported that LNR at the recurrent nerve lymph nodes occurred in only one of 71 patients (1.4%) with T1 esophageal squamous cell carcinoma who received definitive radiotherapy/CRT or adjuvant CRT following ER.^31^ This discrepancy could be explained by the different doses at the ENI, as Uchinami et al used 39.6‐50.4 Gy, which is higher than the 40.0‐41.4 Gy from the present study. Moreover, despite most head and neck cancers (as well as esophageal cancer) being histologically considered squamous cell carcinoma, it is common to use a dose of approximately 50‐55 Gy to the ENI.^32^ Therefore, we propose that a dose of approximately 50 Gy may be more appropriate for sites with a high risk of LNR (eg, the recurrent nerve or perigastric lymph nodes for T1bLVI+ tumors). Dose escalation clearly increases the risks of cardiovascular and gastrointestinal toxicities, although the recurrent nerve lymph nodes are somewhat distant from the heart, and therefore, the risk of cardiovascular toxicities may not grow. Moreover, the risk of toxicity could also be reduced by using newer radiotherapy modalities, such as intensity‐modulated radiotherapy or proton therapy.^33^, ^34^ It may also be possible to consider additional chemotherapy after the adjuvant CRT, as used for locally advanced esophageal cancer.^35^

The present study has several limitations. First, the single‐center retrospective design is associated with inherent selection biases. Second, the small sample size may limit the power of the analyses, and we did not apply multiple testing correction to the analysis of the cumulative incidence of LNR. Furthermore, the risk of LNR in T1b‐SM1 tumors with or without LVI remains unclear since the majority of T1b tumors were considered T1b‐SM2 in the present study. In addition, sex was not considered in the univariate and multivariate analyses, as no female patients developed LNR. However, we confirmed that the risk of LNR in the male patients was also marginally higher for T1bLVI+ tumors than for T1aLVI+ or T1bLVI− tumors (data not shown). Finally, lack of information precluded the consideration of various other factors, including alcohol consumption, smoking habit, and degree of tumor differentiation.

In conclusion, the present study revealed that LNR was relatively common after adjuvant CRT following ER for patients with T1bLVI+ SESCC, and that the recurrent nerve lymph nodes were the most frequent metastasis sites. One of the advantages of combining ER with CRT is the ability to predict the risk of metastasis based on the resected specimen, which may help optimize treatment strategies based on this risk. Therefore, our findings may help guide the development of new strategies for treating SESCC.

## CONFLICT OF INTEREST

None declared.
